# Whole-genome DNA hyper-methylation in iPSC-derived dopaminergic neurons from Parkinson’s disease patients

**DOI:** 10.1186/s13148-019-0701-6

**Published:** 2019-07-23

**Authors:** Rubén Fernández-Santiago, Angelika Merkel, Giancarlo Castellano, Simon Heath, Ángel Raya, Eduard Tolosa, María-José Martí, Antonella Consiglio, Mario Ezquerra

**Affiliations:** 1 Department of Neurology, Lab of Parkinson Disease and Other Neurodegenerative Movement Disorders, Institut d’Investigacions Biomèdiques August Pi i Sunyer (IDIBAPS), Hospital Clínic de Barcelona, Faculty of Medicine (UB), University of Barcelona, Casanova 143, Floor 3B, 08036 Barcelona, Spain; 2 Centro de Investigación Biomédica en Red de Enfermedades Neurodegenerativas (CIBERNED), 28031 Madrid, Spain; 3 Statistical Genomics Team at the Centro Nacional de Análisis Genómico (CNAG-CRG), Centre de Regulacio Genómico (CRG), The Barcelona Institute of Science and Technology, 08028 Barcelona, Spain; 4 Dept. of Anatomic Pathology, Pharmacology and Microbiology, Institut d’Investigacions Biomèdiques August Pi i Sunyer (IDIBAPS), University of Barcelona, 08036 Barcelona, Spain; 5 Center of Regenerative Medicine in Barcelona (CMRB), Hospital Duran i Reynals, Hospitalet de Llobregat, 08908 Barcelona, Spain; 6 Centre for Networked Biomedical Research on Bioengineering, Biomaterials and Nanomedicine (CIBER-BBN), 28029 Madrid, Spain; 7 Institució Catalana de Recerca i Estudis Avançats (ICREA), 08010 Barcelona, Spain; 8 Movement Disorders Unit, Dept. of Neurology, Hospital Clínic de Barcelona, Institut d’Investigacions Biomèdiques August Pi i Sunyer (IDIBAPS), University of Barcelona, 08036 Barcelona, Spain; 9 Department of Pathology and Experimental Therapeutics, Faculty of Medicine, Instituto de Investigación Biomédica de Bellvitge (IDIBELL), University of Barcelona, 08907 Barcelona, Spain; 10 Institute of Biomedicine of the University of Barcelona (IBUB), 08028 Barcelona, Spain; 11 Department of Molecular and Translational Medicine, University of Brescia, 25123 Brescia, Italy

**Keywords:** Parkinson disease (PD), iPSC-derived DAn, DNA methylation, Whole-genome bisulfite sequencing (WGBS), DMCpGs, Differentially methylated CpGs

## Abstract

**Background:**

Parkinson’s disease (PD) is characterized by the loss of midbrain dopaminergic neurons (DAn). Previously, we described the presence of DNA hyper- and hypo-methylation alterations in induced pluripotent stem cells (iPSC)-derived DAn from PD patients using the Illumina 450K array which prominently covers gene regulatory regions.

**Methods:**

To expand and contextualize previous findings, we performed the first whole-genome DNA bisulfite sequencing (WGBS) using iPSC-derived DAn from representative PD subjects: one sporadic PD (sPD) patient, one monogenic LRRK2-associated PD patient (L2PD), and one control.

**Results:**

At the whole-genome level, we detected global DNA hyper-methylation in the PD which was similarly spread across the genome in both sPD and L2PD and mostly affected intergenic regions.

**Conclusion:**

This study implements previous epigenetic knowledge in PD at a whole genome level providing the first comprehensive and unbiased CpG DNA methylation data using iPSC-derived DAn from PD patients. Our results indicate that DAn from monogenic or sporadic PD exhibit global DNA hyper-methylation changes. Findings from this exploratory study are to be validated in further studies analyzing other PD cell models and patient tissues.

**Electronic supplementary material:**

The online version of this article (10.1186/s13148-019-0701-6) contains supplementary material, which is available to authorized users.

## Background

Parkinson’s disease (PD) is a neurodegenerative disorder characterized by motor disabilities due to loss of dopaminergic neurons (DAn) in the substantia nigra pars compacta (SNpc) [1]. Mutations in leucine-rich repeat kinase 2 (*LRRK2*) cause monogenic LRRK2-associated PD (L2PD) and are the most frequent cause of disease [2]. The *LRRK2* p.G2019S variant explains up to 6% familial and 3% sporadic PD (sPD) cases in Europeans [3], but the penetrance is limited [4] suggesting additional factors modifying its expressivity [5]. In this context, epigenetic alterations including DNA methylation changes at CpG sites play a role in neurodegenerative diseases including Alzheimer’s disease [6, 7]. In PD, DNA methylation changes were reported in postmortem brain [7, 8] and blood [9]. An emerging approach for epigenetic investigation of PD involves the use of iPSC-derived dopaminergic neurons (DAn) from PD patients. We recently described aberrant DNA methylation profiles in iPSC-derived DAn from 4 L2PD and 6 sPD patients (total 10 PD vs. 4 controls) [10] which preceded long-term PD phenotypes in PD DAn [11]. In that study, we used the Illumina 450K genome-wide methylation array which interrogates 450,000 CpG in 99% of RefSeq genes but yet representing only 0.02% of the total CpGs in the human genome. To expand and contextualize previous findings here, we selected the samples with the highest epigenetic differences between PD and controls from the previous study which were representative of different PD states (1 L2PD, 1 sPD, and 1 control) and performed a whole-genome DNA bisulfite sequencing (WGBS) analysis. Our study provides the first DNA methylation fingerprint of monogenic and sporadic PD using the DAn cells targeted by disease and genuine to PD live patients [12]. The comprehensive and non-biased DNA methylation data generated in this study is informative for the clinic and for designing future epigenetic research strategies in PD.

## Methods

### Subjects

The Local Ethics Committee at the Hospital Clínic de Barcelona and the Commission on Guarantees for Donation and Use of Human Tissues and Cells (ISCIII) approved the study. Subjects were diagnosed and recruited at the Hospital Clínic de Barcelona after written informed consent [10, 11]. We used 30 days iPSC-derived DAn generated upon reprogramming of skin fibroblasts into iPSC and differentiation into DAn. Detailed protocol [13] and DAn cell line characterization of study subjects are described previously [10, 11]. Based on array findings in the 14 subjects of study [10], we selected for WGBS analysis one representative subject per condition including 1 L2PD male patient (44 years old), 1 sPD female patient (51 years old), and 1 female control (66 years old) (Additional file 1: Table S1).

### Whole-genome bisulfite sequencing and library construction

A total of 2 μg of genomic DNA was spiked with unmethylated bacteriophage *λ* DNA (5 ng of *λ* DNA per microgram of genomic DNA; Promega) and methylated T7 phage DNA (5 ng of T7 DNA per microgram of genomic DNA). DNA was shared by sonication to 50–500 bp using a LE220 Focused-ultrasonicator (Covaris). Fragments of 150–300 bp were size-selected using AMPure XP beads (Agencourt Bioscience). Genomic DNA libraries were constructed using the Illumina TruSeq Sample Preparation kit following Illumina’s protocol. After adaptor ligation, DNA was treated with sodium bisulfite using the EpiTect Bisulfite Kit (Qiagen), following the manufacturer’s instructions. Two rounds of bisulfite conversion were performed to ensure conversion rates above 99%. Enrichment for adaptor-ligated DNA was done through seven PCR cycles using the PfuTurboCx Hotstart DNA polymerase (Stratagene). Library quality was monitored using the Agilent 2100 Bioanalyzer, and concentrations were estimated by KAPA Library Quantification Kit Illumina® Platforms (Kapa Biosystems). Paired-end DNA sequencing (2 × 101 bp) of converted libraries was performed using the HiSeq2000 (Illumina) following the manufacturer’s protocol with HiSeq Control Software (HCS-1.5.15.1). Average sequencing depth was similar and around 30 for all subjects (27 for L2PD, 32 for sPD, and 31 for the control). Images analysis, base calling, and quality scoring of runs were processed using the software Real Time Analysis (RTA-1.13.48) followed by generation of FASTQ sequence files. Sequencing data were deposited in the European Genome-phenome Archive (EGA) at the Centre for Genomic Regulation (CRG) under accession Nr. EGAD00001003922.

### WGBS data processing and identification of differentially methylated CpGs

Short read alignment and methylation estimation were perform using the gemBS analysis pipeline [14]. Single CpG quantitative methylation values were calculated as the ratio of unconverted reads to the sum of unconverted and converted reads. We used the GRCh38/hg38 genome version as reference. CpG sites from each sample were filtered based on a genotype calling quality threshold (minimum PHREAD-scaled genotype score of 20) to eliminate sites that possibly contained SNPs. In total, 23,796,355 autosomal CpG sites passed the filters in all three samples, and this set was used for the subsequent analyses. DMCpG were identified from the multiple pairwise comparison of CpG sites from all 3 subjects, selecting sites where the absolute difference in the methylation estimates was above 0.25 with a *p* value below 1 × 10^−8^. The *p* value was calculated using the approach of Raineri et al. [15], which calculates the exact probability that two methylation probabilities, estimated from the ratio of non-converted to converted bases, differ given the number of converted and non-converted bases in the two samples. In all cases, hyper- and hypo-methylation were defined relative to the levels in the control sample. We defined “uniquePD” as all differentially methylated sites detected in L2PD vs. control and sPD vs. control, but not in L2PD vs. sPD, i.e., DMCpGs involved in the common PD pathogenic process. Annotation of DMCpGs to gene-related regions [10] and to functional chromatin states [16] (epidermal keratinocyte NHEK-E127) data was done as earlier reported. In addition, to compare methylation levels between WGBS and the Illumina 450K array, we calculated correlation coefficients by performing a Spearman correlation analysis as requested for non-normal bimodal DNA methylation data.

### Biological enrichment analysis

To determine whether genes associated with DMCpGs in sPD or L2PD were enriched in particular gene ontology (GO) terms, we used the Webgestalt software [17] and adjusted the *p* values by the Benjamini and Hochberg FDR multiple testing correction [18].

## Results

**Fig. 1:**
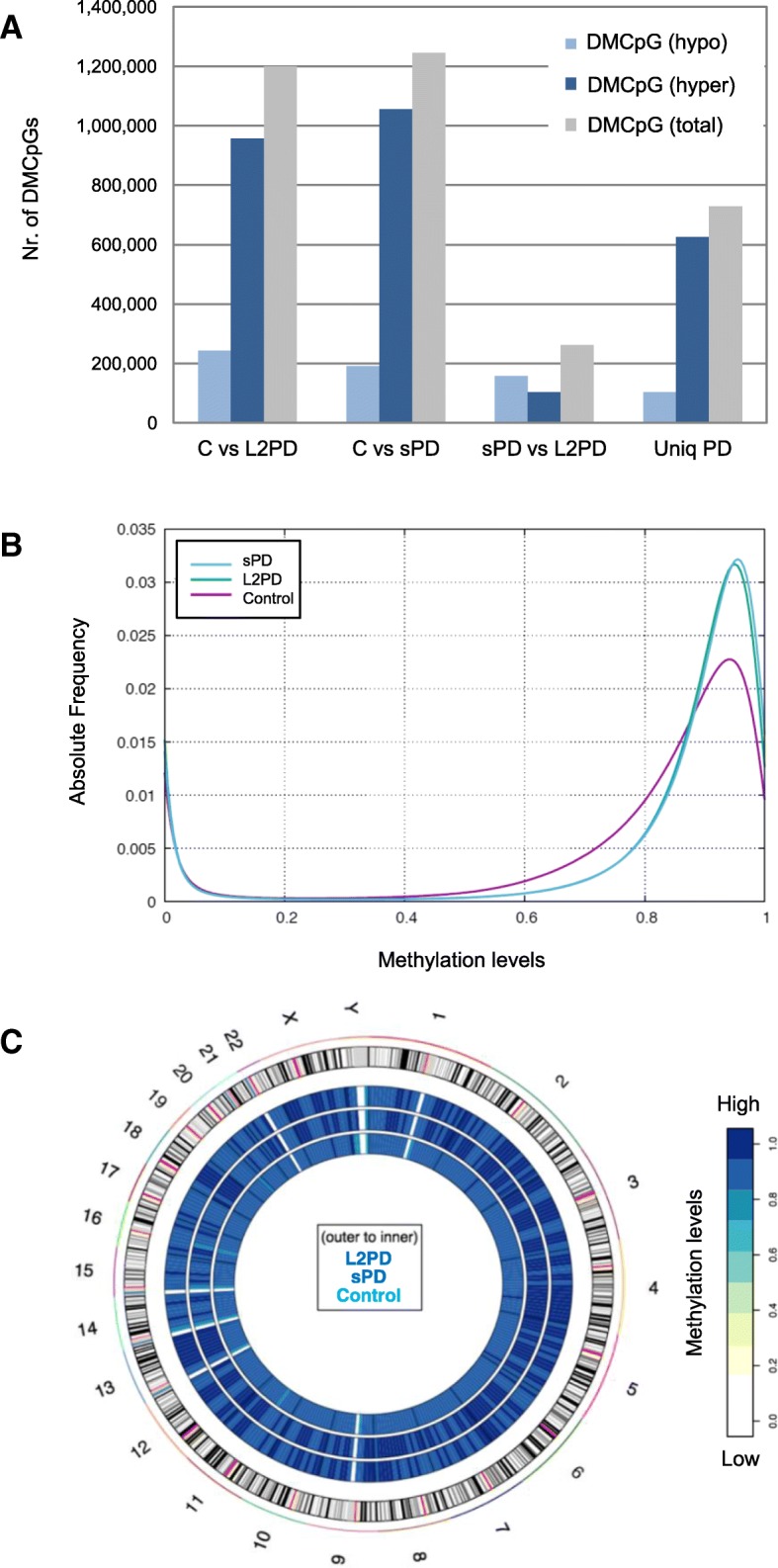
DMCpGs detected by whole-genome bisulfite sequencing in iPSC-derived DAn from L2PD, sPD, and control subjects. **a** Total of hypo-methylated (light blue) and hyper-methylated (dark blue) DMCpGs and total DMCpGs (grey). Unique PD (Uniq PD) comprises PD-specific DMCpGs detected in L2PD vs. control and in sPD vs. controls but not in L2PD vs. sPD. **b** Histogram of absolute frequencies of overall CpG methylation levels in study subjects. **c** Circus plot of whole-genome DNA methylation changes in study subjects. The outer circle displays an ideogram ordered by chromosome number

We analyzed the WGBS data from the L2PD, sPD, and control selected subjects under the standard cutoff of a methylation difference above 25% and a *p* < 1 × 10^−8^. We observed 1,199,391 differentially methylated CpGs (DMCpGs) in L2PD vs. control (5.04%) and 1,245,691 DMCpGs in sPD vs. control (5.04%) (Fig. 1a). Per contrary, we only found 261,459 DMCpGs in L2PD vs. sPD (1.10%) indicating little difference between PD cases. Most of the identified DMCpGs, i.e., 729,216 (60%), were common in both PDs. Moreover, the PD-associated changes consisted of a large DNA hyper-methylation in L2PD and sPD (Fig. 1b, c) (79.8% in L2PD and 84.7% in sPD) whereas hypo-methylation was up to 5-fold less frequent. In addition, the PD-specific hyper-methylation was spread throughout the genome. Altogether, these results indicate a commonly shared methylation deregulation in monogenic L2PD and sPD consisting in a global DNA hyper-methylation.

**Fig. 2:**
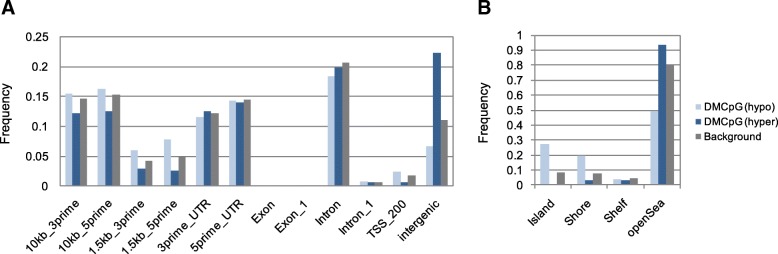
Genomic and functional annotation of DMCpGs in L2PD and sPD**.** Both CpGs analyzed by WGBS and 450K array data were annotated using the UCSC Genome Browser database (hg19). Relative distribution of frequencies of unique DMCpGs (i.e., unique and common to L2PD and sPD) across different gene-related regions (**a**) and GpG island context (**b**), showing hypo-methylated DMCpGs (light blue) and hypo-methylated DMCpGs (dark blue) compared to the background

To expand and contextualize previous findings, we compared the WGBS data from selected subjects with the 450K data of the entire cohort (*n* = 4 L2PD, *n* = 6 sPD, *n* = 4 controls) [10]. Over 96% of CpG sites in the 450K array overlapped with the analyzed set of CpGs from WGBS. We found an overall high correlation of findings between array and WGBS (Spearman’s *r* = 0.955 for L2PD, 0.951 for sPD, and 0.922 for control) (Additional file 2: Figure S1). Out of the 1261 DMCpGs identified by array in L2PD vs. controls, we found 1116 high-quality CpGs by WGBS (88.5%) of which 891 (79.8%) were DMCpGs associated with L2PD (Additional file 1: Table S2). From the 2512 array DMCpGs in sPD vs. controls, we detected 2281 CpGs by WGBS (90.8%) of which 1812 (79.9%) were DMCpGs associated with sPD. In addition to the PD enhancer hyper-methylation reported earlier at the gene level using the array [10], at the whole-genome level here we observed a prominent enrichment of hyper-methylated DMCpGs located at intergenic non-coding regions, in CpG low regions (“open sea”), and distant from CpG islands (Fig. 2a, b). Overall, the WGBS data largely overlapped but expanded previous array data at the whole-genome level by showing an intergenic hyper-methylation which is associated with monogenic L2PD and sPD.

Finally, we explored the molecular functions regulated by the PD-associated DMCpGs. To this end, we selected the 5000 most variable DMCpGs across all samples identified between PD (L2PD and sPD) and controls (Additional file 3: Table S3). Of these, 25% DMCpGs were mapped to intergenic regions whereas the remaining 75% DMCpGs were annotated to genes. The biological enrichment analysis of these genes indicated that the PD-associated methylation largely targeted genes involved in neural functions (Table 1). These whole-genome methylation results are in line with previous epigenetic findings at the gene-related context [10].

**Table 1:** Gene ontology enrichment analysis of genes annotated to the 5000 top-end statistically most significant unique DMCpGs identified in IPSC-derived DAn from sPD and L2PD patients

Code	Biological processes	*p* value	FDR
GO:0006928	Movement of cell or subcellular component	6.66E−16	6.06E−12
GO:0040011	Locomotion	5.33E−14	2.42E−10
GO:0022008	Neurogenesis	1.58E−13	4.78E−10
GO:0048646	Anatomical structure formation involved in morphogenesis	2.66E−12	4.38E−09
GO:0048870	Cell motility	2.89E−12	4.38E−09
GO:0051674	Localization of cell	2.89E−12	4.38E−09
GO:0007417	Central nervous system development	4.33E−12	5.49E−09
GO:0009887	Animal organ morphogenesis	5.11E−12	5.49E−09
GO:0030182	Neuron differentiation	5.58E−12	5.49E−09
GO:0048666	Neuron development	6.04E−12	5.49E−09
Code	Cellular component	*p* value	FDR
GO:0097458	Neuron part	1 < E−17	1 < E−17
GO:0031226	Intrinsic component of plasma membrane	1 < E−17	1 < E−17
GO:0005887	Integral component of plasma membrane	1 < E−17	1 < E−17
GO:0044463	Cell projection part	1 < E−17	1 < E−17
GO:0120038	Plasma membrane-bounded cell projection part	1 < E−17	1 < E−17
GO:0043005	Neuron projection	1 < E−17	1 < E−17
GO:0098590	Plasma membrane region	1 < E−17	1 < E−17
GO:0045202	Synapse	1 < E−17	1 < E−17
GO:0044456	Synapse part	1 < E−17	1 < E−17
GO:0098794	Postsynapse	1 < E−17	1 < E−17
Code	Molecular function	*p* value	FDR
GO:0008092	Cytoskeletal protein binding	2.34E−07	3.42E−04
GO:0004653	Polypeptide N-acetylgalactosaminyltransferase activity	4.93E−07	3.42E−04
GO:0004714	Transmembrane receptor protein tyrosine kinase activity	7.34E−07	3.42E−04
GO:0001012	RNA polymerase II regulatory region DNA binding	8.79E−07	3.42E−04
GO:0019199	Transmembrane receptor protein kinase activity	9.94E−07	3.42E−04
GO:0000977	RNA polymerase II regulatory region sequence-specific DNA binding	1.25E−06	3.42E−04
GO:0005088	Ras guanyl-nucleotide exchange factor activity	1.28E−06	3.42E−04
GO:0005085	Guanyl-nucleotide exchange factor activity	2.56E−06	6.00E−04
GO:0019904	Protein domain-specific binding	3.30E−06	6.87E−04
GO:0005509	Calcium ion binding	3.85E−06	7.22E−04

Enrichment *p* values were adjusted by FDR multiple testing correction

## Discussion

Here, we report the first WGBS study using iPSC-derived DAn from PD patients. At the whole-genome level, we found a PD-associated methylation deregulation consisting of a global DNA hyper-methylation common in monogenic L2PD and sPD. Our findings agree with previous studies showing that despite subtle differences [19], L2PD uniquely resembles sPD at the clinical and neuropathological level [20] where the variant p.G2019S causes not only L2PD but also sPD without familial segregation. Moreover, despite both PD forms can be initiated by different mechanisms [21], L2PD and sPD were earlier shown to share common epigenetic, transcriptomic, and microRNA alterations [10, 22]. In addition, the PD-associated hyper-methylation targeted genes involved in neural functions as reported previously [5], but in fact, DMCpGs were enriched in intergenic regions. Although we found a high degree of correlation between the previous data using the Illumina 450K array and the present study, this large DNA hyper-methylation was not previously reported. This was probably due to the design of the array which mostly focuses on CpGs located at gene-related regions. The precise function of intergenic non-coding regions are only starting to be uncovered, but these regions could play a role in pathogenic processes of human disease by affecting transcription regulatory regions or non-coding transcripts as lncRNAs, miRNAs, siRNAs, piRNAs, and snoRNAs [23], thus potentially contributing to regulating gene expression of other genes.

In PD animal models, increased global methylation was proposed as a cause for parkinsonism leading to DA depletion, hypokinesia, and tremor [24]. Yet global PD hyper-methylation can be compatible with hypo-methylation at specific promoters as recently shown in PD blood and cortex [7]. At the gene level, we previously reported a deficit of transcription factors (TF) in PD DAn relevant to dopaminergic differentiation which was associated with PD enhancer hyper-methylation [10]. It is possible that a deficiency of TFs may mediate genomic hyper-methylation in specific genomic regions in PD DAn. One possibility is that the global hyper-methylation detected in PD at the whole genome level might be related to functional imbalances in the enzymatic machinery regulating DNA methylation such as DNA methyltransferases (DNMTs) or DNA demethylases. In this regard, DNMTs have been shown to be involved in neural differentiation [25] and recent studies reported genetic association of DNMTs variants with PD [26, 27] and their altered expression in PD postmortem brain [28]. Yet elucidating the biological significance of the identified PD genomic hyper-methylation and role of intergenic regions is out of scope of the current report and requires further investigation.

We previously demonstrated the presence of epigenetic alterations in iPSC-derived DAn from PD [5]. Here, we performed a complete characterization of CpG methylation changes in these cells but the small sample size and the potential confounder effect of age and gender are a limitation of this study. In addition, the fact that the reprogramming process from fibroblast to iPSC in itself involves epigenetic modifications could also represent a confounder effect. Yet, our study provides the first DNA methylation fingerprint using iPSC-derived DAn from PD patients by showing a global DNA hyper-methylation spread across the genome which is similar in monogenic L2PD and sPD. This comprehensive and unbiased data may be informative for the clinic to design future epigenetic research approaches in PD. Future studies validating our findings also in PD patient tissues or in PD cell models and also elucidating the functional role of hyper-methylation in PD are warranted.

## Conclusions

iPSC-derived DAn from PD patients exhibit global DNA hyper-methylation changes associated with disease. This hyper-methylation is common to the monogenic L2PD and the sporadic forms of disease. Our study highlights the importance of performing WGBS as to implement array-based studies to provide an accurate and comprehensive methylation picture of the PD epigenome.

## Additional files

Additional file 1: **Table S1.** Clinic demographic features of study subjects and iPSC-derived DAn cell lines characterized by whole-genome bisulfite sequencing (WGBS). Keynote: (a) Initial symptom (T, tremor; B, bradykinesia). (b) Ratio of neurons/total cells as estimated by immunofluorescence as the ratio of TUJ1 (neuron-specific class III b-Tubulin)-positive cells/ DAPI-positive cells. (c) Ratio of iPSC-derived DAn/total neurons estimated by immunofluorescence as the ratio of TH (tyrosine hydroxylase)-positive cells/TUJ1-positive cells. N/A, not assessed. **Table S2.** Number of CpGs detected as differentially methylated CpGs (DMCs) using the Infinium Human Methylation 450K BeadChip Kit using iPSC-derived DAn from 6 sPD, 4 L2PD, and 4 healthy controls in the previous study (Fernandez-Santiago et al., 2015). Of these, number and percentage of CpGs genotyped, and detected as DMCs by whole genome bisulfite sequencing (WGBS) at minimum quality 30 read coverage using representative samples using iPSC-derived DAn from one representative sample per group (1 L2PD, 1 sPD, and 1 healthy control). (DOCX 16 kb)

Additional file 2: **Figure S1.** CpGs methylation plots and Spearman’s correlation coefficients for pairwise comparisons between WGBS and Illumina 450K high-density arrays (450,000 CpG methylation sites at single base resolution). (PDF 74 kb)

Additional file 3: **Table S3.** List of the top 5000 DMCs in L2PD, sPD, and uniquePD. (XLSX 2247 kb)

## Data Availability

The datasets supporting the conclusions of this article are included within the article and its additional files, and complete sequencing data were deposited in the European Genome-phenome Archive (EGA) at the Centre for Genomic Regulation (CRG) under accession Nr. EGAD00001003922. The datasets generated during the current study are not publicly available due to the ethical implications of performing a complete genome sequencing of subjects but are available from the corresponding author on reasonable request.

## Abbreviations

DA: Dopamine
DAn: Ddopaminergic neurons
DMCpGs: Ddifferentially methylated CpGs
iPSC: Iinduced pluripotent stem cells
L2PD: *LRRK2*- associated PD
PD: Parkinson’s disease
sPD: Ssporadic PD
WGBS: Wwhole-genome bisulfite sequencing
