# Supplementary material for: Whole-genome DNA hyper-methylation in iPSC-derived dopaminergic neurons from Parkinson’s disease patients
Source: Clin Epigenetics. 2019 Jul 23;11:108. doi: 10.1186/s13148-019-0701-6 (PMC6651999; doi:10.1186/s13148-019-0701-6)
Supplement: Supplementary file 1 — Table S1. Clinic demographic features of study subjects and iPSC-derived DAn cell lines characterized by whole-genome bisulfite sequencing (WGBS). Keynote: (a) Initial symptom (T, tremor; B, bradykinesia). (b) Ratio of neurons/total cells as estimated by immunofluorescence as the ratio of TUJ1 (neuron-specific class III b-Tubulin)-positive cells/ DAPI-positive cells. (c) Ratio of iPSC-derived DAn/total neurons estimated by immunofluorescence as the ratio of TH (tyrosine hydroxylase)-positive cells/TUJ1-positive cells. N/A, not assessed. Table S2. Number of CpGs detected as differentially methylated CpGs (DMCs) using the Infinium Human Methylation 450K BeadChip Kit using iPSC-derived DAn from 6 sPD, 4 L2PD, and 4 healthy controls in the previous study (Fernandez-Santiago et al., 2015). Of these, number and percentage of CpGs genotyped, and detected as DMCs by whole genome bisulfite sequencing (WGBS) at minimum quality 30 read coverage using representative samples using iPSC-derived DAn from one representative sample per group (1 L2PD, 1 sPD, and 1 healthy control). (DOCX 16 kb) [file 13148_2019_701_MOESM1_ESM.docx]

**Additional file 1. Table S1.** Clinic demographic features of study subjects and iPSC-derived DAn cell lines characterized by whole-genome bisulfite sequencing (WGBS). Keynote: (a) Initial symptom (T, tremor; B, bradykinesia); (b) Ratio of neurons / total cells as estimated by immunofluorescence as the ratio of TUJ1 (neuron-specific class III b-Tubulin)-positive cells/ DAPI-positive cells; (c) Ratio of iPSC-derived DAn / total neurons estimated by immunofluorescence as the ratio of TH (tyrosine hydroxylase)-positive cells / TUJ1 positive cells; N/A: not assessed,

| Code previous study 1 | Code previous study 2 | Subject type | *LRRK2* mutation | Family history  of PD | Gender | Age at donation | Age at onset | (a)  Initial symptoms | L-DOPA response | (b)  Cell ratio TUJ1^+^ / DAPI^+^ (neurons) | (c)  Cell ratio TH^+^ / TUJ1^+^  (DAn) |  |
| --- | --- | --- | --- | --- | --- | --- | --- | --- | --- | --- | --- | --- |
| C-03 | SP-09 | Control | No | No | Male | 66 | - | - | - | 52.2 | 55.5 |  |
| PD-04 | SP-16 | sPD | No | No | Female | 51 | 48 | B | N/A | 32.1 | 55.2 |  |
| PD-05 | SP-06 | L2PD | G2019S | Yes | Male | 44 | 33 | T | Good | 40.9 | 61.9 |  |
|  |  |  |  |  |  |  |  |  |  |  |  |  |

**Study 1**: Fernandez-Santiago R, Carballo-Carbajal I, Castellano G, et al. Aberrant epigenome in iPSC-derived dopaminergic neurons from Parkinson's disease patients. EMBO Mol Med 2015:7(12):1529-1546. // **Study 2**: Sanchez-Danes A, Richaud-Patin Y, Carballo-Carbajal I, et al. Disease-specific phenotypes in dopamine neurons from human iPS-based models of genetic and sporadic Parkinson's disease. EMBO Mol Med 2012;4(5):380-395.

**Additional file 1. Table S2.** Number of CpGs detected as differentially methylated CpGs (DMCs) using the Infinium Human Methylation 450K BeadChip Kit using iPSC-derived DAn from 6 sPD, 4 L2PD, and 4 healthy controls in the previous study (Fernandez-Santiago et al., 2015), Of these, number and percentage of CpGs genotyped, and detected as DMCs by whole genome bisulfite sequencing (WGBS) at minimum quality 30 read coverage using representative samples using iPSC-derived DAn from one representative sample per group (1 L2PD, 1 sPD, and 1 healthy control).
