# Supplementary material for: Whole-genome DNA hyper-methylation in iPSC-derived dopaminergic neurons from Parkinson’s disease patients
Source: Clin Epigenetics. 2019 Jul 23;11:108. doi: 10.1186/s13148-019-0701-6 (PMC6651999; doi:10.1186/s13148-019-0701-6)
Supplement: Supplementary file 2 — Figure S1. CpGs methylation plots and Spearman’s correlation coefficients for pairwise comparisons between WGBS and Illumina 450K high-density arrays (450,000 CpG methylation sites at single base resolution). (PDF 74 kb) [file 13148_2019_701_MOESM2_ESM.pdf]

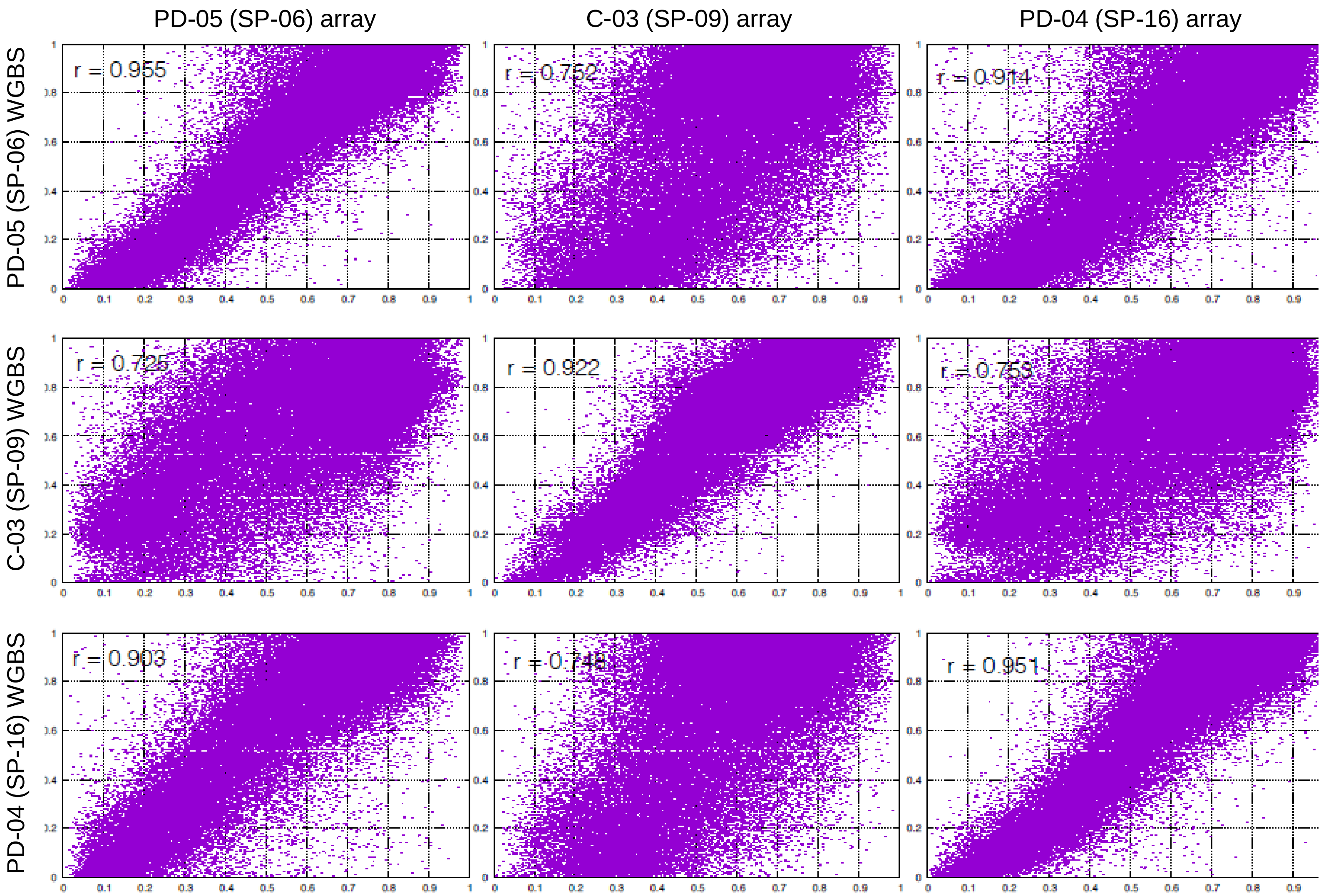

Figure S1. CpGs methylation plots and Spearman correlation coefficients for pairwise comparisons between WGBS and Illumina 450k high-density arrays (450,000 CpG methylation sites at single base resolution).
